# Nonlinear inelastic electron scattering from Au nanostructures induced by localized surface plasmon resonance

**DOI:** 10.1038/s41598-018-24065-z

**Published:** 2018-04-04

**Authors:** ZheAn Li, ChunKai Xu, WenJie Liu, Meng Li, XiangJun Chen

**Affiliations:** 1 Hefei National Laboratory for Physical Sciences at the Microscale and Department of Modern Physics, University of Science and Technology of China, Hefei, 230026 China; 2 Synergetic Innovation Center of Quantum Information and Quantum Physics, University of Science and Technology of China, Hefei, 230026 China

## Abstract

Nonlinear electron scattering is a recently-discovered physical process observed during the localized plasmonic excitation of Ag nanostructures on graphite surface. In the present work, nonlinear electron scattering phenomena is experimentally verified on Au nanostructures by measuring inelastic scattering of electrons field-emitted from tungsten tip. The relative intensity of the electron-energy-loss peak associated with the plasmonic excitation of Au shows again to increase nonlinearly with the electric field generated by the tip-sample bias, demonstrating the generality of nonlinear electron scattering process in plasmonic system. Compared to the nonlinear electron scattering phenomena observed on Ag nanostructures, the nonlinear term for Au nanostructures is about 1 to 2 orders of magnitude smaller, which is in consistent with the field enhancement factor of Au and Ag nanostructures from both the surface-enhanced Raman spectroscopy experiments and the theoretical calculations.

## Introduction

Scanning probe electron energy spectroscopy (SPEES) is an emerging technique which can obtain spectroscopy mapping of surface with spatial resolution^1–8^. In this technique, the tip of a scanning tunnel microscope is operated in the field emission mode to generate a local electron beam, which can be scattered from the surface and collected by an electron energy analyzer. Therefore, the two dimensional distribution of the electron energy spectra of the surface can be scanned^2,5,7,8^, which is important information for fully understanding the surface at microscale. However, the probability of the inelastic scattering of electron is usually very small, often orders of magnitude smaller than that of the elastically scattered electron. It thus becomes a bottleneck for improving the spatial resolution of this developing technique. This difficulty may be overcome by a recently-discovered new physical process, named nonlinear electron scattering, in which the intensity of the inelastic scattering electron signal can be nonlinearly enhanced to the same level of its elastic counterpart, during the excitation of the localized surface plasmon resonance (LSPR) on Ag nanostructures^9^. According to the theoretical explanation^9^, extremely high localized electric field is produced by the “hot spots” formed between Ag nanostructures and modulated by the external electric field, which consequently induces nonlinear electron scattering process. This is benefited from the large field enhancement factor of Ag nanostructures, as often demonstrated in the surface-enhanced Raman spectroscopy (SERS) experiments^10–17^. On the other hand, Au nanostructure also has large field enhancement factor, which, in combination with its high chemical and physical stability, makes it being widely used in the panorama of nanoscience and nanotechnology^18–20^. Therefore, it should be very interesting to investigate whether the large field enhancement factor of Au nanostructures can also lead to the nonlinear electron scattering phenomena.

In this article, we report the SPEES experiments carried out on Au nanostructures on graphite surface. It is observed that the relative intensity of the inelastic scattered electron due to the Au LSPR excitation increases nonlinearly with respect to the external electric field generated by the tip-sample bias, demonstrating the generality of the nonlinear electron scattering phenomena in plasmonic system during the electron scattering process. The observed nonlinear term for Au nanostructures is about 1 to 2 orders of magnitude smaller than that for Ag nanostructures. This is in accordance with the field enhancement factor of Au and Ag nanostructures obtained by the SERS experiments^21–24^, as well as the theoretical calculations^25^.

## Results

**Figure 1:**
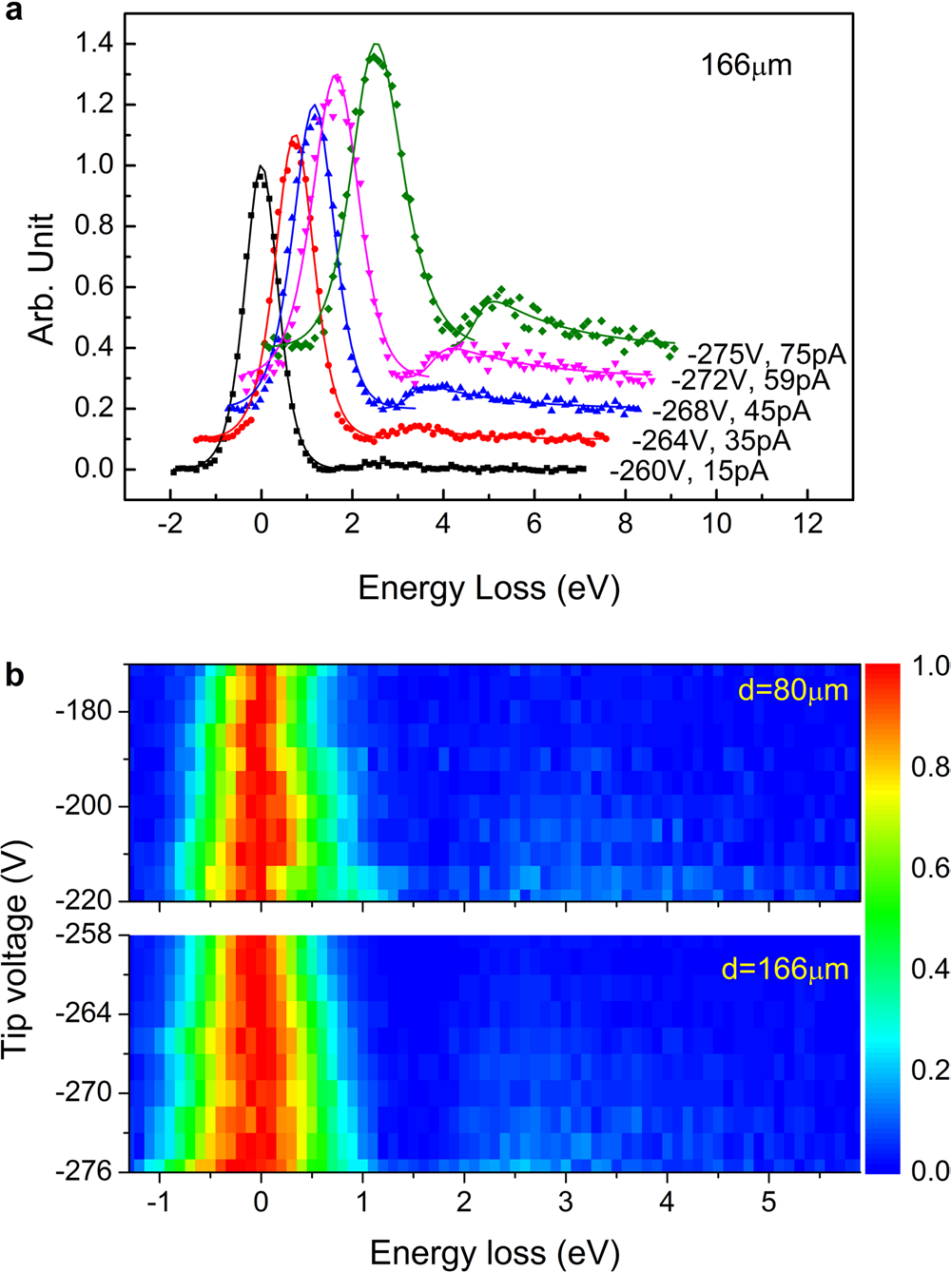
EELSs acquired at different tip voltages under different tip-sample distances. (**a**) EELSs acquired at tip-sample distance 166μm with different tip voltages and sample currents. (**b**) Two dimensional plots of the measured electron scattering intensity as a function of energy loss and tip voltage at two different tip-sample distances. For comparison, all EELSs have been background-subtracted by polynomial function and divided by the amplitude of the elastic scattering peak.

The experiments are carried out by a home-made scanning probe electron energy spectrometer, the detailed description of which can be found elsewhere^5^. Briefly, it consists of a tip-sample system and a toroidal electron energy analyzer (TEEA)^26^. In the experiment, electrons field-emitted from a tungsten tip are used to excite the LSPR of Au nanostructures, which is prepared by evaporating 30 nm Au on HOPG surface. The scattered electrons are collected and analyzed by the TEEA (see Methods). Electron energy loss spectra (EELS) are obtained at two different tip-sample distances, 166 μm and 80 μm. At each distance, the tip voltage is increased step by step. As examples, EELS acquired at 166 μm with five different tip voltages are shown in Fig. 1(a), where the experimental data are denoted as scattered points and the fitted curves are plotted as solid lines. Each spectrum has been background-subtracted by polynomial function and normalized by the amplitude of the elastic scattering peak for comparison^4,9^. The energy loss peak located at around 2.6 eV is attributed to the excitation of Au LSPR. Similar to the phenomena observed on Ag nanostructures^9^, the intensity of the LSPR peak in EELS obtained at relatively low tip voltage is very weak, whereas it is significantly enhanced when the tip voltage increases. The full data acquired at two different tip-sample distances are presented by two dimensional plots in Fig. 1(b), showing the electron scattering intensity as a function of energy loss and tip voltage. It can be seen that at both tip-sample distances, the intensity of the LSPR peak increases with the tip voltage.

## Discussion

**Figure 2:**
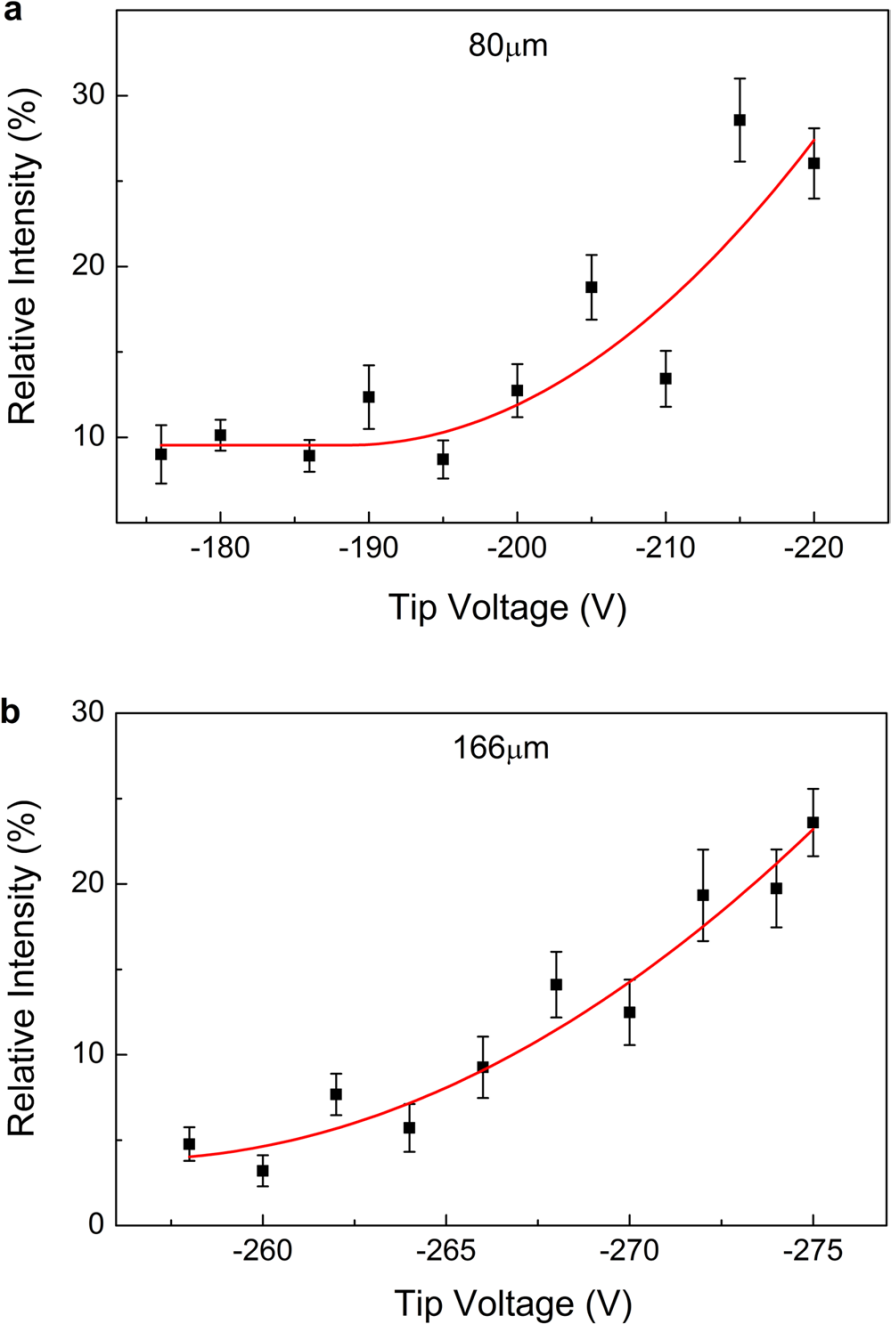
The dependence of the relative intensity (*RI*) on tip voltage under different tip-sample distances. (**a**) 80 μm, (**b**) 166 μm. The solid square with error bar represents calculated *RI* from the experimental data, and the solid lines are quadratic fitted curves.

From the surface electronic excitation theory^27–29^, we have the knowledge that surface plasmon is excited mainly through the long-range dipole scattering process. At backscattering condition in our experiments, the LSPR excitation must be a multiple-scattering process of a large-angle elastic scattering followed by a near-zero-angle dipole scattering. Therefore the transition probability of LSPR excitation can be obtained experimentally from the intensity ratio of the inelastic scattering peak to the elastic scattering peak in EELS, which is defined as the relative intensity (*RI*) in our previous work on Ag nanostructures^9^. Similarly, in this work we also obtain *RI* for Au nanostructures. Its dependence on tip voltage at each tip-sample distance is shown in Fig. 2. It is obvious that *RI* increases nonlinearly with the tip voltage, demonstrating the occurrence of the nonlinear electron scattering process during the LSPR excitation on Au nanostructures. However, the *RI* increase rate with respect to tip voltage are smaller than that of Ag nanostructures, which should be attributed to the difference between the properties of the LSPR states for Au nanostructures and Ag nanostructures.

According to the theory proposed in ref.^9^, the transition probability *W*_*ba*_ of the LSPR excitation can be described by the perturbation theory considering the second-order interaction under the dipole approximation, and can be expressed as

1 $${W}_{ba}={W}_{ba}^{(1)}+{W}_{ba}^{(2)}\propto {|\langle b|{\overrightarrow{E}}_{1}\cdot {\overrightarrow{D}}_{ba}|a\rangle |}^{2}+\sum _{n}{|\frac{\langle b|{\overrightarrow{E}}_{2}\cdot {\overrightarrow{D}}_{bn}|n\rangle \langle n|{\overrightarrow{E}}_{1}\cdot {\overrightarrow{D}}_{na}|a\rangle }{{{\rm{\Gamma }}}_{n}/2}|}^{2}$$

Here $|a\rangle$ and $|b\rangle$ are the ground state and the excited LSPR state, and $|n\rangle$ is an intermediate state with energy width ${\Gamma }_{n}$. ${\overrightarrow{D}}_{ba}$, ${\overrightarrow{D}}_{bn}$ and ${\overrightarrow{D}}_{na}$ are the matrix elements of the dipolar moment operator for electron scattering, ${\overrightarrow{E}}_{1}$ and ${\overrightarrow{E}}_{2}$ are the electric field experienced by $|a\rangle$ and $|n\rangle$, and the sum over *n* is over all possible intermediate states. In the conventional EELS experiments such as high resolution EELS (HREELS)^29,30^ and EELS in scanning transmission electron microscope (STEM-EELS)^31–34^, the second-order term ${W}_{ba}^{(2)}$ is much smaller than the first-order term ${W}_{ba}^{(1)}$ and can be neglected, consequently the transition probability is dominated by the latter one and shows no dependence on the external field. However, in the nonlinear electron scattering phenomena observed in the present work on Au nanostructures as well as in the previous work on Ag nanostructures^9^, the probability of the LSPR excitation is largely enhanced, indicating that ${W}_{ba}^{(2)}$ should play an important role. From the electromagnetic mechanism in SERS experiments^21–25^, we have the knowledge that noble metal nanostructure will enhance the incoming electric field by a large field enhancement factor *f* through LSPR, generating an extremely high localized electric field. The same mechanism can also be applied to electron scattering process, resulting in an enhanced local field when the LSPR of noble metal nanostructure is excited. According to the expression

2 $${W}_{ba}^{(2)}\propto {|\frac{\langle b|{\overrightarrow{E}}_{2}\cdot {\overrightarrow{D}}_{bn}|n\rangle \langle n|{\overrightarrow{E}}_{1}\cdot {\overrightarrow{D}}_{na}|a\rangle }{{{\rm{\Gamma }}}_{n}/2}|}^{2}$$

all intermediate states $|n\rangle$ can in principle contribute to ${W}_{ba}^{(2)}$. However, as described in ref.^9^, only the final state $|b\rangle$ will experience the enhanced field and thus contribute mostly to ${W}_{ba}^{(2)}$, which is then approximated to

3 $${W}_{ba}^{(2)}\propto {|\frac{\langle b|{\overrightarrow{E}}_{2}\cdot {\overrightarrow{D}}_{b}|b\rangle \langle b|{\overrightarrow{E}}_{1}\cdot {\overrightarrow{D}}_{ba}|a\rangle }{{{\rm{\Gamma }}}_{b}/2}|}^{2}\propto {(\frac{2{\mu }_{b}{E}_{2}}{{{\rm{\Gamma }}}_{b}})}^{2}{W}_{ba}^{(1)}.$$

Here *μ*_*b*_ and Γ_*b*_ are permanent dipole moment and energy width of the final state $|b\rangle$ respectively, and ${\overrightarrow{E}}_{2}$ is the enhanced localized electric field. This will lead to a significant LSPR peak in EELS, and the transition probability of LSPR, i.e. *RI*, should be quadratically dependent on the electric field, as clearly shown by our experimental observations on both Au nanostructures and Ag nanostructures. The coefficient of the quadratic term (can also be referred to nonlinear coefficient) should consequently be proportional to ${f}^{2}$. Therefore, comparing the nonlinear coefficient of Au nanostructures with that of Ag nanostructures will provide information about the field enhancement factor for LSPR state, which is very important in the field of plasmonics and nanophotonics.

From the tip-sample distance and tip voltage, we can estimate the electric field, and the dependence of *RI* on electric field can be obtained. By fitting the data with a quadratic function, the nonlinear coefficient can be determined. The results for the two tip-sample distances of Au nanostructures are listed in Table 1. The nonlinear coefficients for Ag nanostructures at three different tip-sample distances are also determined from the previous nonlinear electron scattering experimental data^9^ and compiled in Table 1 for comparison.

**Table 1:** The nonlinear coefficient.

	Au		Ag^9^		
tip-sample distances (μm)	80	166	92	114	150
nonlinear coefficient (μm^2^/V^2^)	1	15	166	67	83

It is clearly shown that the nonlinear coefficient for Au nanostructures is about 1 to 2 orders of magnitude smaller than that for Ag nanostructures. The field enhancement factor *f* for Ag and Au nanostructures has been studied intensively by both SERS experiments^21–24^ and theoretical calculations^25^. Orendorff *et al*. measured the electromagnetic enhancement factor (EF) of SERS for Ag and Au nanostructures with average size about 30 nm^21^, which are quite similar to the samples we used in our experiment. By measuring SERS signal from 4-Mercaptopyridine molecules deposited on surface, the EFs for Ag and Au nanostructures are determined to be 4.8 ± 0.5 × 10^6^ and 1.2 ± 0.2 × 10^4^ respectively. Considering EF is proportional to ${f}^{4}$^15^, their results are in good agreement with our measurements. On the other hand, Tanabe^25^ directly calculated the field enhancement factors for Ag and Au nanoparticles, which is also in consistent with our experiments. From these comparisons, it is evidently confirmed that nonlinear electron scattering should be a general phenomenon existing in plasmonic system. The results also indicate that the nonlinear electron scattering is a potential method to study the properties of the LSPR states of noble metal nanostructures, which is very important but difficult to be investigated due to the ultra-short dumping time.

In summary, EELSs for Au nanostructures on HOPG surface have been measured by a home-made scanning probe electron energy spectrometer with different tip voltages at different tip-sample distances. It is shown that the relative intensity *RI* of the inelastic scattering electron due to Au LSPR excitation increases nonlinearly with the electric field, demonstrating the generality of nonlinear electron scattering process in plasmonic system. Compared to the previous nonlinear electron scattering work on Ag nanostructures, the nonlinear coefficient for Au nanostructures is about 1 to 2 orders of magnitude smaller, which is in consistent with previous studies on field enhancement factors for Au and Ag nanostructures by both SERS experiments and theoretical calculations.

As we have mentioned in our last paper^9^, the observation of nonlinear electron scattering lays the foundation for a new spectroscopic technology, namely nonlinear electron scattering spectroscopy (NESS). The confirmation of the generality of the nonlinear electron scattering phenomenon in this work further augments the possibility of this potential technology. Due to the high chemical and physical stability of Au, it would be more proper to employ Au nanostructures as the substrate in NESS technology to generate localized electric “hot spot”, which will act as a highly confined ultra-fast laser source^35,36^ to excite the adsorbates and consequently high spatial and spectral resolution will be acquired.

## Methods

### Experimental setup

**Figure 3:**
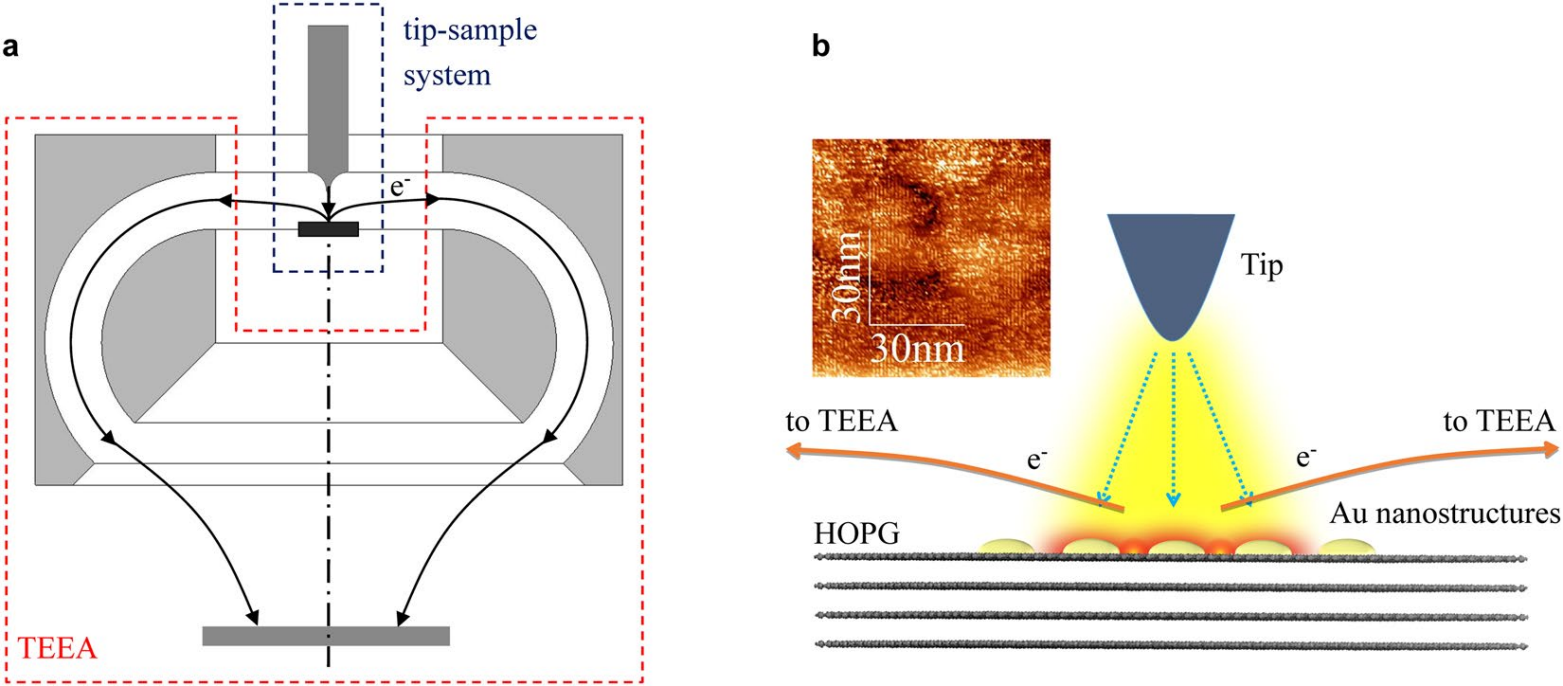
The experimental arrangement. (**a**) The sketch of the spectrometer which consists of a tip-sample system and a toroidal electron energy analyzer (TEEA). (**b**) The enlarged part of the tip-sample area. A tungsten tip is approached to a distance of micrometers from the sample surface, which is prepared by evaporating about 30 nm thick Au on HOPG. Au nanostructures can be observed in the topography image as shown in the inset. A negative voltage of hundred volts is applied to the tip while keep the sample surface grounded to produce field-emission electrons. The localized surface plasmon resonance of the Au nanostructures is excited by the field-emission electrons and the backscattered electrons are collected and energy-analyzed by a TEEA.

The experiments are carried out by a home-made scanning probe electron energy spectrometer, detailed description of which can be found elsewhere^5^. Briefly, it consists of a tip-sample system and a toroidal electron energy analyzer (TEEA)^26^, and is capable of acquiring EELS under a close tip-sample distance with different tip voltages. The geometry of the experiment is illustrated in Fig. 3a, while Fig. 3b shows the enlarged part of the tip-sample area. In the experiment, a tip made from a 0.42 mm tungsten wire by electrochemical etching was approached to a distance of micrometers from the grounded sample surface, which was prepared by evaporating 30 nm thin film of Au on freshly cleaved HOPG. The topography image of the sample surface is shown in the inset of Fig. 3b, where Au nanostructures can be observed. A negative voltage *V*_*t*_ of hundred volts was then applied to the tip to generate field-emission electrons to impact perpendicularly with the sample surface. According to the geometry of the spectrometer shown in Fig. 3a, the backscattered electrons outgoing parallel to the sample surface were collected and analyzed by the TEEA. In this way the EELS under a certain tip-sample distance and tip bias was acquired. In the present work, the experiments were performed at two tip-sample distances, 166 μm and 80 μm.

### Data analysis

For each EELS, firstly the background-subtraction by polynomial function was performed. Then we deconvoluted the elastic scattering (ES) peak and the LSPR excitation peak in EELS, and calculate the *RI* by dividing the LSPR peak area with ES peak area. By increasing the tip voltage step by step while keeping the tip-sample distance unchanged, the EELSs at different electric field were obtained, and consequently the corresponding *RI*s were calculated.

### Data availability

The data that support the findings of this study are available from the corresponding author (C.K.X.) upon reasonable request.
